# Ethylmalonic encephalopathy and liver transplantation: long-term outcome of the first treated patient

**DOI:** 10.1186/s13023-021-01867-5

**Published:** 2021-05-19

**Authors:** Giorgia Olivieri, Diego Martinelli, Daniela Longo, Chiara Grimaldi, Daniela Liccardo, Ivano Di Meo, Andrea Pietrobattista, Anna Sidorina, Michela Semeraro, Carlo Dionisi-Vici

**Affiliations:** 1 Division of Metabolism, Bambino Gesù Children’s Hospital, IRCCS, Piazza S. Onofrio 4, 00165 Rome, Italy; 2 Neuroradiology Unit, Bambino Gesù Children’s Hospital, IRCCS, Rome, Italy; 3 Division of Abdominal Transplantation and Hepatobiliopancreatic Surgery, Bambino Gesù Children’s Research Hospital IRCCS, Rome, Italy; 4 Division of Hepatology and Gastroenterology, Bambino Gesù Children’s Research Hospital IRCCS, Rome, Italy; 5 Unit of Medical Genetics and Neurogenetics, Fondazione IRCCS Istituto Neurologico Carlo Besta, Milan, Italy

**Keywords:** Liver transplantation, Ethylmalonic encephalopathy, *ETHE1*, Thiosulphate, Motor function

## Abstract

**Background:**

Ethylmalonic encephalopathy (EE) is a severe intoxication-type metabolic disorder with multisystem clinical features and leading to early death. In 2014, based on the promising results obtained by liver-targeted gene therapy in *Ethe1*^*−/−*^ mouse model, we successfully attempted liver transplantation in a 9-month-old EE girl. Here we report her long-term follow-up, lasting over 6 years, with a comprehensive evaluation of clinical, instrumental and biochemical assessments.

**Results:**

Neurological signs initially reverted, with a clinical stabilization during the entire follow-up course. Accordingly, gross motor functions improved and then stabilized. Psychomotor evaluations documented an increasing communicative intent, the acquisition of new social skills and the capability to carry out simple orders. Neurophysiological assessments, which included EEG, VEP/ERG and BAEPs, remained unchanged. Brain MRI also stabilized, showing no further lesions and cerebral atrophy improvement. Compared to pre-transplant assessments, urinary ethylmalonic acid strikingly reduced, and plasma thiosulphate fully normalized. The child maintained good clinical conditions and never experienced metabolic crises nor epileptic seizures.

**Conclusions:**

The long-term follow-up of the first EE transplanted patient demonstrates that liver transplantation stabilizes, or even improves, disease course, therefore representing a potentially elective option especially in early-diagnosed patients, such as those detected by newborn screening, before irreversible neurological damage occurs.

## Background

Ethylmalonic Encephalopathy (EE) was first described in 1991 as a severe multisystem disorder, characterized by early-onset encephalopathy, petechial purpura, orthostatic acrocyanosis, chronic diarrhea and progressive spasticity, biochemically associated with ethylmalonic aciduria, elevated C4- and/or C5- acyl-carnitine esters, and hyperlactacidemia [1, 2]. Some years later, *ETHE1* was identified as the causative gene. *ETHE1* encodes for a protein targeted into mitochondria [3], whose function was then elucidated in playing a key role in hydrogen sulphide (H_2_S) detoxification as a sulphur dioxygenase [4]. This newly discovered metabolic pathway is ubiquitously expressed in humans, and it is involved in the detoxification of H_2_S, mainly produced by anaerobic bacteria in the large intestine and, to a lesser extent, by sulphurated aminoacids catabolism in peripheral tissues [5]. Therefore, *ETHE1* mutations cause sulphur dioxygenase deficiency, which lead to the toxic accumulation of H_2_S and its metabolites in tissues and body fluids, making EE an “intoxication type” metabolic disease [6]. H_2_S and its stable derivative thiosulphate have a strong vasoactive and vasotoxic effect [7], causing a widespread multisystem endothelial vascular damage, mainly located in brain, skin, muscle and gastrointestinal tract [8]. Moreover, H_2_S accumulation exerts an inhibitory effect on several mitochondrial enzymes, including cytochrome C oxidase (COX) and short chain acyl-CoA dehydrogenase (SCAD), thus explaining the characteristic biochemical abnormalities [3, 4]. Secondary COX deficiency mainly affects energetic metabolism in brain and muscle, causing lactic acidosis, stroke-like encephalopathic crises, epileptic seizures and myopathy [8], while SCAD inhibition causes the elevation of ethylmalonic acid and of C4- and C5- acyl-carnitine esters [1, 2].

Few studies reported the potential beneficial effects of a combined treatment with N-acetylcysteine (NAC), metronidazole (MTZ), and the dietary restriction of the sulphur containing aminoacid methionine in decreasing serum thiosulphate and ameliorating the clinical course (Table 1) [9–11]. However, these approaches have a limited impact on the natural disease history in patients with severe EE [12]. In this regard, a spectrum of clinical severity exists in EE, which includes, beside the severe form presenting with multisystem clinical features and leading to early death, a handful of cases with an “atypical” mild phenotype, characterized by pyramidal dysfunction, minor cognitive involvement, no systemic signs and longer survival [13–15].

**Table 1:** Outcome in EE patients treated by liver transplantation compared to medical therapies (follow-up ≥ 1 year)

Reference	Genotype	Age at onset	Phenotype	Age at last follow-up	Liver Transplant age	Low protein diet start–end	NAC therapy start–end	MTZ Therapy start–end	EMA baseline/follow-up	Reported outcome at follow-up
Present Case	c.131_132delAG/131_132delAG	7 m	Severe	7 y	9 m		7 m–ongoing	7 m–21 m	205/40	Disease stabilization, achievement of cognitive and social skills (says few words, sits unaided), persistence of diarrhea and skin signs, MRI improvement of atrophy
[28]	del exon 4/del exon 4	NBS	Severe	2.3 y	19 m		13 m–ongoing	13 m–ongoing		Improvement of social and language skills, sits unaided, babbling
[28]	c.487C>T/c.487C>T	NBS	Severe	2.9 y	13 m		2 m–ongoing	2 m–ongoing	153/31	Seven months after transplant episode of metabolic decompensation, then slow improvements in motor, verbal and social skills (sit unaided, says few simple words)
[29]	c.375 + 5G>A/c.462 T>A	1 m	Severe	3.2 y	18 m	15 m–18 m		15–20 m	179/70.7	No remarkable general amelioration, disease stabilization, quadriplegia, achievement of few words, head control, persistence of skin signs and diarrhea, MRI stabilization
[11]	c.131_132delAG/c.566delG	NBS	Severe	2.1 y		8 m–ongoing	1.5 m–ongoing	1.5 m–ongoing	617.7/383.9	Slow developments in motor and verbal skills, sits unaided, says few words, mild skin signs, no diarrhea
[32]	c.448G>A/c.448G>A	10 m	Severe	2.5 y		15 m–ongoing	15 m–ongoing	15 m–ongoing		Disease stabilization, psychomotor achievements, says simple words, walks with aid
[11]	c.505 + 1G>A/c.505 + 1G>A	NBS	Severe	2.1 y			2 m–ongoing	2 m–ongoing	586/352.2	Sits unaided, poor growth, diffuse skin signs, non-verbal achievement, good visual attention, no diarrhea
[15]	c.79C>A/c.79C>A	10 y	Mild	19 y			16 y–ongoing	16 y–ongoing	72/74	Improvement of mobility and speech, at 17 years episode of acute decompensation with seizures followed by regain of functionalities
[31]	c.3G>T/c.3G>T	5 y	Mild	15 y			11 y–ongoing	11 y–ongoing	46/24	Improved diarrhea and QoL, worsening of paraparesis, normal IQ, normal MRI

*EMA* Ethylmalonic acid, mean values expressed as mmol/mol creatinine, *MTZ* metronidazole, *NAC N*-acetylcysteine, *NBS* newborn screening, *QoL* quality of life

In 2012, a liver-targeted gene therapy was attempted in recombinant *Ethe1*^−/−^ mouse model with encouraging results. This innovative approach, based on restoring the expression of *ETHE1* in the liver, allowed a marked increase of hepatic ETHE1 function and the clearance of most of the circulating H_2_S from intestinal blood. As a fact, thiosuphate levels decreased to nearly normal levels with a significant improvement in treated animals’ survival time [16].

On this basis, we successfully attempted a living-donor orthotopic liver transplantation in a 9 month-old child with the “classical” severe EE phenotype [17]. Within the first year of follow-up, her biochemical abnormalities promptly reversed and psychomotor development improved. Here we report her long-term post-transplant follow-up, lasting over 6 years, with a comprehensive description of impact of liver transplantation on clinical, neurophysiological, neuroradiological and biochemical outcomes.

## Case report

### The patient

**Fig. 1:**
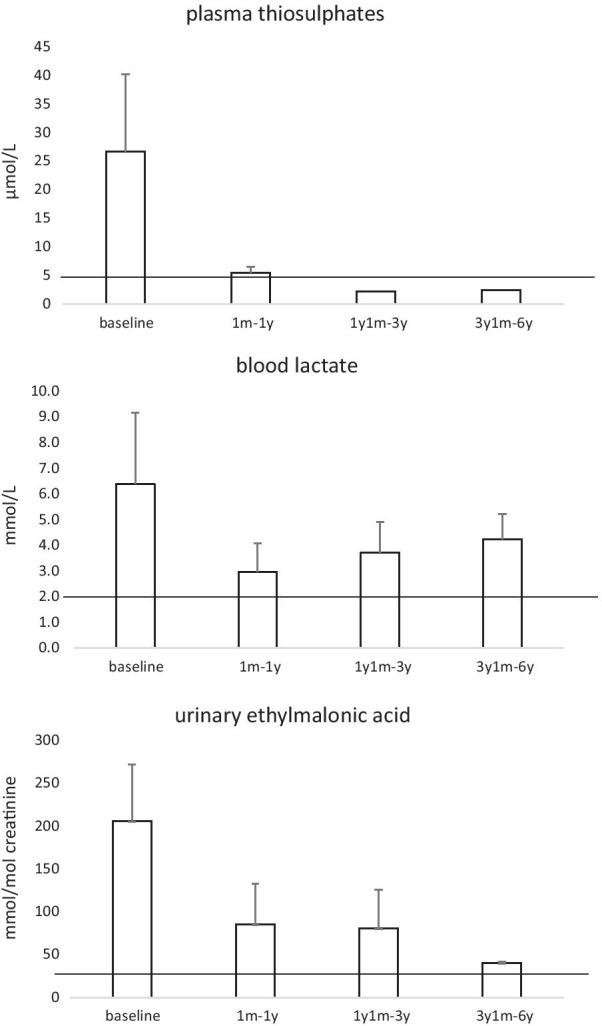
Longitudinal biochemical assessments. Plasma thiosuphates and urinary ethylmalonic acid showed a striking and stable improvement after liver transplantation, reaching values close to normal levels; blood lactate values showed a reduced trend. Solid lines indicate the upper reference values

**Fig. 2:**
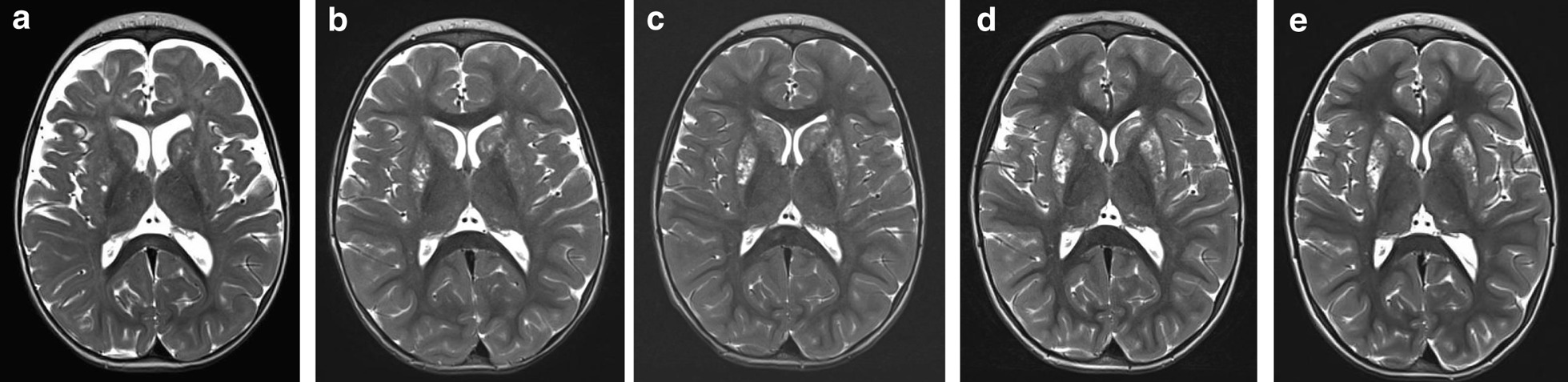
Longitudinal brain MRI findings. Brain MRI T2-weighted sections before living transplantation (**a**), and at one (**b**), two (**c**), three (**d**) and five (**e**) years of follow-up. Before liver transplantation, fronto-temporal atrophy and symmetric hyperintense lesions involving the putamina and the caudate nuclei were detectable (**a**). After liver transplantation, improvement of cerebral atrophy with no others new basal ganglia lesions occurred (**b**–**e**). A slight progression of basal ganglia lesions, especially at the left putaminal level, was observed between the 1st and the 3rd year (**b**–**d**), and then stabilized (**d–e**)

Clinical and biochemical data about the first year of follow-up have been previously reported [17]. Briefly, the child was referred to the Metabolic Unit of Bambino Gesù Hospital in Rome at the age of 7 months, as she was diagnosed with EE due to *ETHE1* homozygous c.131-132delAG mutation. The clinical picture included moderate psychomotor delay, axial hypotonia and lower limb spasticity, associated to severe petechial purpura and orthostatic acrocyanosis. Biochemically she presented elevation of C4- and C5-acylcarnitines, plasma thiosulphate and urinary ethylmalonic acid (Fig. 1). Brain MRI showed basal ganglia involvement and mild cortical atrophy (Fig. 2a). Treatment with NAC (100 mg/kg/day), MTZ (30 mg/kg/day) and carnitine (100 mg/kg) was rapidly started. With the aim to improve the natural course of the disease, based on the promising results obtained by liver-targeted gene therapy in the mouse model, she underwent liver transplantation at the age of 9 months, by receiving the left lobe from her heterozygous mother.

## Methods

After transplantation, the patient underwent longitudinal clinical and neurological evaluations. Motor function assessment were performed by the Gross Motor Function Measure 66-item version (GMFM-66) [18]. Psychomotor development was assessed by the Mental Developmental Griffiths' Scales (MDGS). Neurophysiological studies included electroencephalogram (EEG), visual evoked potentials (VEPs) with electroretinogram (ERG), and brain steam auditory evoked potentials (BAEPs). Target metabolites, consisting of plasma thiosulphate and urinary ethylmalonic acid, were measured by HPLC and gas-chromatography/mass-spectrometry respectively. Neuroradiological assessments by brain MRI were performed at 1, 2, 3 and 5 years of follow-up. Post-trasplantation data were compared to pre-transplantation ones, in order to verify the effect of liver transplantation on the natural history of the disease.

## Results

### General clinical course

The child maintained good clinical conditions throughout  over 6 year of follow-up. Petechial purpura and orthostatic acrocyanosis persisted, while metabolic crises and epileptic seizures never occurred. Her swallowing function remained unchanged and, to date, she is orally fed with her parent's help. She receives standard immunosuppressive treatment with tacrolimus, antioxidant therapy with NAC (100 mg/kg/day) and low-dose carnitine supplementation (30 mg/kg/day). After transplantation, no dietary restrictions have been applied. To avoid resistances and potential side effects, the initial pre-transplant bowel decontamination therapy with MTZ alone was shifted to a poly-therapy, consisting in the weekly rotation of different antibiotics (i.e. cefixime, ciprofloxacin, rifaximin and nystatin). As for chronic diarrhea, we observed a fluctuating ameliorating trend in terms of stool frequency when courses with different antibiotics included cefixime and ciprofloxacin, which were therefore mainly maintained.

Bicarbonate supplementation was started to correct mild metabolic acidosis, likely related to the side effects of immunosuppressive treatment with tacrolimus on kidney function.

### Neuromotor assessment

**Fig. 3:**
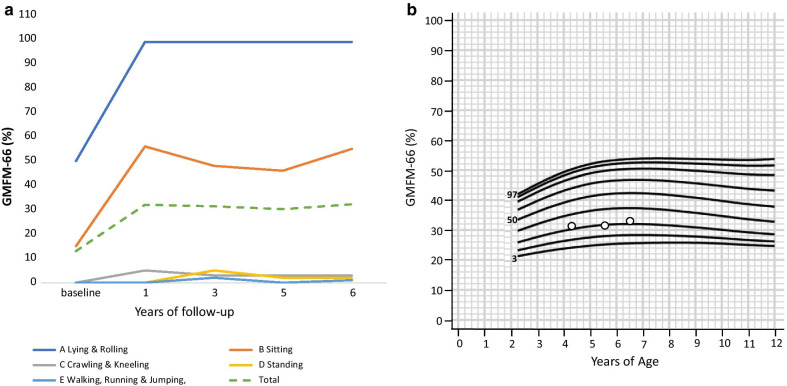
Motor function abilities. **a** Motor function outcome according to the different domains. **b** Motor function outcome during follow-up, according to the Gross Motor Function Classification System (GMFCS) Table of percentile—Level IV [21]

Before liver transplantation, the patient presented axial hypotonia, pyramidal signs at lower limbs and the inability to sit unsupported. Within the first 6 months after transplantation, she progressively reached new motor skills, including the ability to sit unaided, push herself up to her forearms and roll over. At 2 years of follow-up she was able to maintain static standing with an external support, as long as she was placed in standing position with leg braces. Up to the fourth year of follow-up, she was able to walk forward with an external support at upper limbs. At the same time, under an intensive rehabilitation program, she strengthened other motor skills, improving motor functions at the trunk and upper limbs. Consistent with the clinical outcome, GMFM-66 total score rapidly improved from pre-transplantation value of 12–20% and 32%, respectively 6 months and one year later, and then stabilized, scoring 31% at 3 years (12°p), 30% at 5 years (9°p), 32% at 6 years (10°p) (Fig. 3).

### Cognitive assessment

Longitudinal MDGS scored a mental age of 10 months versus 21 months of chronological age at 1 year of follow-up, and of 17 months versus 4.2 years of chronological age at 3 years of follow-up, consistent with a severe psychomotor delay. Further cognitive evaluations were later attempted at 5 and 6 years of follow-up. At these times, the increasing gap between the test requests accorded to the chronological age and the objectivable performances make not possible to obtain standardized scores. Manual skills resulted grossly affected by peripheral muscle weakness, and the absence of verbal language hampered to accurately quantify the child's level of comprehension. However, at the 5th year of follow-up, she presented a very good visual interaction and the capability to carry out simple one-step orders (e.g. “give me that toy” or “move that object from here to there”) and at the 6th year she showed a good communicative intent, mainly focused on gesture, and the capability to perform simple cognitive tasks (e.g. recognizing the main colors or some animals) with slight improvements in verbal skills as well, allowing to pronounce few simple words (e.g. “mom”, “water”, etc.).

### Neurophysiological studies

VEPs/ERG and BAEPs resulted normal at baseline and remained unchanged at follow-up controls, which were performed annually. Awake-EEG showed a slight and diffuse slowdown of the background activity over years, while epileptic paroxysmal were never recorded.

### Neuroradiological findings

Brain MRI performed before transplantation documented a mild brain atrophy and initial bilateral involvement of basal ganglia. After liver transplantation, longitudinal MRI assessment showed a progressive reduction of periencephalic spaces up to complete resolution of brain atrophy. With regard to basal ganglia, a very slight progression of the left putamen lesion occurred between the 1st and the 3rd year of follow-up, and then stabilized (Fig. 2).

### Biochemical results

Before liver transplantation, plasma thiosulphate levels were grossly elevated (26.8 ± 13.5 µmol/L; nv < 4.0). Afterwards, we observed a rapid ameliorating trend, with a complete and stable normalization at follow-up (range 2.2–2.4 µmol/L). Consistently, urinary ethylmalonic acid values dropped from 205.8 ± 66.2 mmol/mol creatinine (nv < 20.0) to 40.0 ± 1.4 mmol/mol creatinine at follow-up. Blood lactate values also reduced from 6.4 ± 2.8 mmol/L (nv < 2.2) to 4.3 ± 1.0 mmol/L (Fig. 1).

## Discussion

This report shows that liver transplantation stabilized the long-term disease course in a severe case of EE, thus making this therapeutic option a suitable approach in EE, as seen in other intoxication type metabolic diseases [19, 20]. The liver transplant effectiveness in EE relies on restoring hepatic deficient sulphur dioxygenase activity, thereby providing the hepatic filtering support to clear most of the circulating H_2_S, grossly produced in the gastro intestinal tract [7]. Our patient presented a homozygous frameshift mutation (c.131–132delAG), responsible for a premature biallelic stop codon at the *ETHE1* second exon, with deleterious impact on protein expression [17].

Consistent with the genotype, she presented the “classical” severe form of EE, with a very poor neurological outcome and a short life expectancy. However, her clinical course was significantly modified by liver transplantation associated with maintenance of standard medical therapy, which allowed clinical improvement and long-lasting stabilization. Different from the usual disease course, she never experienced metabolic crises nor epileptic seizures, while became able to achieve relevant improvements in both motor and cognitive skills. At the age of 4 years, when she reached her higher motor function ability, she was classified, according to the Gross Motor Function Classification System (GMFCS) [21], as having a level IV severity disability. At a longer-term follow-up, her gross motor function curve stabilized, remaining within the same percentile trajectory. This trend is consistent with a static neurological disease, such as an infantile cerebral palsy [21], and is by far very different from what observed in severe EE [12]. Accordingly, neurophysiological assessments documented the integrity of the visual and auditory networks, and EEG recorded no epileptic paroxysmal, which is different from the natural course of the diseases [8, 22–25]. Post-transplant brain MRI documented the progressive resolution of cortical atrophy, along with a transient slight progression of a left putaminal lesion, which later stabilized.

Biochemically, plasma thiosuphate, the primary metabolic biomarker of EE, fully normalized, and urinary excretion of ethylmalonic acid markedly reduced up to an almost complete normalization. Blood lactate levels mildly improved as well. The lower impact of liver transplantation on skin and gastrointestinal manifestations, suggests that not only the circulating level of H2S (and of its derivative thiosulphate) contributes to the multi-system clinical phenotype. It is likely that also the local production of toxic compounds in different tissues maintains unchanged some disease symptoms [26]. MTZ therapy was discontinued one year after transplantation due to patient's intolerance (abdominal pain), to avoid antibiotic resistance, for a better effect on chronic diarrhea obtained with different molecules and to prevent the potential side effect on peripheral nerve when used for prolonged periods at high doses [27].

Following our original report [17], three further EE patients underwent liver transplantation [28, 29]. Tam et al*.* [28] reported on two children with severe EE, identified by newborn screening and transplanted at the age of 19 and 13 months respectively, when their neurological picture was already compromised. The first patient, carrying a homozygous deletion of exon 4 of *ETHE1*, received orthotropic liver transplantation from a cadaveric donor and was reported to show a clear improvement of his neurological status over the first nine months after transplantation. The second one, carrying a homozygous severe pathogenic variant in *ETHE1* (c.487C>T, p.R163W), had an elder affected sibling who died at the age of 2 after an encephalopathic episode triggered by a viral infection. After transplantation, a neurological improvement occurred, but seven months later, in the context of a viral gastroenteritis, he presented a severe encephalopatic crisis with a metabolic stroke requiring intubation. Brain MRI showed new extensive lesions in cerebellum, white matter, bilateral striatum and corpus callosum. Nevertheless, at 22 months of follow-up, his clinical outcome was reported to be stable. After transplantation both patients continued NAC and MTZ, and one carnitine therapy. Biochemically, they showed a reduction of urinary excretion of isobutyryl- and 2-methylbutyryl-glycine, with no changes in plasma ethylmalonic acid values in one. Post-transplant data on the primary biomarker plasma thiosulphate and on urinary ethylmalonic acid were not reported in the study [28]. A further EE patient, compound heterozygous for a known pathogenic mutation (c.375 + 5G>A) and a novel mutation (c.462T>A, p.D154E) in *ETHE1*, received liver transplant with a reduced-size left lateral lobe donated by his heterozygous mother at the age of 18 months [29]. Before transplant the neurological examination revealed marked axial hypotonia, muscle weakness, increased deep tendon reflexes, clonus, and bilateral Babinski sign with fronto-temporal atrophy and multiple bilateral symmetrical lesions of basal ganglia at brain MRI. During 20 months of follow-up, brain MRI suggested a certain improvement in basal ganglia lesions. Clinically the patient still presented developmental delay and neurologic disability, with a mild amelioration of petechiae and ecchymosis and without improvement of chronic mucoid diarrhea and orthostatic acrocyanosis. After transplantation, MTZ and carnitine therapy was initially continued with clear improvement of metabolite levels. Subsequently, metronidazole was interrupted due to patient’s intolerance resulting in the increase of urinary EMA levels while 2-methylsuccinic acid levels gradually restored to normal.

The long-term outcome in the four patients treated by liver transplantation in comparison with those receiving medical therapies (i.e. diet, antioxidant and decontaminating drugs) with a follow-up ≥ 1 year is shown in Table 1. Despite an overall limited experience, liver transplantation improved or stabilized the disease course in at least two out of four patients. As seen in other metabolic diseases, liver transplantation although not allowing to fully cure the disease may impact favorably on its course [30]. After transplantation, metronidazole (or intestinal antibiotic therapy) and carnitine therapy is advisable to further improve the metabolic profile by reducing the major offending metabolites. Taken together, these data suggest that liver transplantation should be considered an elective therapeutical option in EE, especially in early diagnosed patients, and in particular in those identified by newborn screening, before irreversible neurological damages occur. For a better understanding of the impact of liver transplantation on EE, a multidisciplinary approach in the long-term management of disease-targeted therapies, immunosuppressive regimen, and in monitoring biochemical and neurological outcomes is advisable.

## Conclusions

This case history, reporting the long-term follow-up in the first transplanted severe EE patient, confirms that liver transplantation represents the most effective therapeutic option to be considered in early diagnosed EE cases, before the occurrence of progressive and irreversible neurological damages.

## Data Availability

All data generated and analysed during this study are included in this published article.
